# Heterogeneous treatment effects of a text messaging smoking cessation intervention among university students

**DOI:** 10.1371/journal.pone.0229637

**Published:** 2020-03-05

**Authors:** Marcus Bendtsen

**Affiliations:** Department of Health, Medicine and Caring Sciences, Linköping University, Linköping, Sweden; University of Mississippi Medical Center, UNITED STATES

## Abstract

**Introduction:**

Despite tobacco being an important preventable factor with respect to ill health and death, it is a legal substance that harms and kills many of those who use it. Text messaging smoking cessation interventions have been evaluated in a variety of contexts, and are generally considered to have a positive effect on smoking cessation success. In order for text messaging interventions to continue to be useful as prevalence of smoking decreases, it may be necessary to tailor the interventions to specific individuals. However, little is known with regard to who benefits the most and least from existing interventions.

**Methods:**

In order to identify heterogenous treatment effects, we analyzed data from a randomized controlled trial of a text messaging smoking cessation intervention targeting university students in Sweden. We used a Bayesian hierarchical model where the outcome was modelled using logistic regression, and so-called horseshoe priors were used for coefficients. Predictive performance of the model, and heterogeneous treatment effects, were calculated using cross-validation over the trial data.

**Results:**

Findings from the study of heterogenous treatment effects identified less effect of the intervention among university students with stronger dependence of nicotine and students who smoke a greater quantity of cigarettes per week. No heterogeneity was found with respect to sex, number of years smoking, or the use of snuff.

**Discussion:**

Results emphasize that individuals with a more developed dependence of nicotine may have a harder time quitting smoking even with support. This questions the dissemination and development of text messaging interventions to university students in the future, as they may not be the optimal choice of intervention for those with a more developed dependence. On the other hand, text messaging interventions may be useful to disseminate among university students that are at risk of developing a strong dependence.

**Trial registration:**

International Standard Randomized Controlled Trial Number (ISRCTN): 75766527; http://www.controlled-trials.com/ISRCTN75766527.

## Introduction

Among the factors considered in the Global Burden of Diseases, Injuries, and Risk Factors Study [1], smoking ranks number two when ordered by risk-attributable disability adjusted life years. Some of the non-communicable diseases for which the risk is higher among smokers include cardiovascular and respiratory diseases, diabetes, and cancer. Despite tobacco being an important preventable factor with respect to ill health and death [2], it is a legal substance that harms and kills many individuals when used as intended by manufacturers [3].

In Sweden, a steady decline in smoking prevalence has been recorded over the past decade by the Public Health Agency of Sweden [4]. The most recent data from 2018 indicate that the prevalence rate is as low as 7% in the general population. This means that we are closer than ever to eradicating one of the most important causes of disease in Sweden. However, adolescents and young adults still start smoking, and many will continue smoking into adulthood with a more severe nicotine dependence. There is therefore still a need for effective smoking cessation interventions that can scale to a national level and that are designed to reach adolescents and young adults.

One promising approach to help young individuals quit smoking is to use text messaging [5–7]. Text messaging interventions typically consist of a series of messages sent to participants’ mobile phones over the course of 8–12 weeks. These messages motivate participants to make a quit attempt and then reinforce and support this decision throughout the intervention period. In addition, text messaging interventions may also increase access to education and support services that promote smoking cessation [6].

Several randomized controlled trials (RCTs) have been conducted to estimate the effect of text messaging interventions for smoking cessation [8–11], notably the txt2stop trial [9] (n = 5800), which found strong evidence in favor of the intervention with respect to both biochemically verified abstinence (OR 2.20, 95% CI 1.80–2.68, P-value < .0001) and self-reported abstinence (OR 1.47, 95% CI 1.40–1.66, P-value < .0001). Three meta-analyses have concluded that text messaging interventions have a positive effect on smoking cessation: one reported a summary effect size of 0.25 (95% CI 0.13–0.38) [5], the second meta-analysis reported an overall summary OR of 1.37 (95% CI 1.25–1.51) of smoking cessation in favor of text messaging interventions [6], and the third analysis similarly found that quit rates were higher among those who had access to text messaging interventions (OR 1.36, 95% CI 1.23–1.51) [7]. Thus, there exists a relatively strong body of evidence for the average treatment effect of text messaging interventions.

As is often the case in RCTs, the effects of text messaging interventions are generally assessed at the group level, which is evident when inspecting the individual trials included in meta-analyses [5–7]. However, we do expect some individuals to benefit more than others from an intervention, and some may even be harmed, as not all individuals with access to smoking cessation interventions successfully quit smoking. Treatment effects may therefore be considered heterogeneous within a group.

As prevalence rates of smoking in Sweden decrease [4], we may find that interventions need to become more targeted towards groups that persist with smoking despite current policy and interventions. It is therefore important to identify who benefits the most and least from existing interventions. While subgroup analyses are sometimes included in reports, these are problematic as they may fail to find effects in smaller subgroups, subgroups may be arbitrarily defined, and they are susceptible to false positives due to multiple hypothesis testing and confounding [12–16]. We therefore need to take a different approach and make predictions which estimate the outcome for an individual in both settings being contrasted. To do this, we need to create a prediction model from the data that we have collected in an RCT, and then assess this model’s performance in order for any findings relating to heterogenous treatment effects to be useful.

This report communicates the results from a study of heterogenous treatment effects that aimed to identify which university students benefit the most and the least from a Swedish text messaging smoking cessation intervention called NEXit. The intervention consists of a 1- to 4-week motivational phase, during which participants can commit to a quit date, followed by a 12-week program with supportive and reinforcing text messages (a total of 157 messages). Data for the included study was taken from the NEXit trial, which estimated the average treatment effect of NEXit among Swedish university students in an RCT [11,17].

## Material and methods

The NEXit trial was a 2-arm randomized trial of a text messaging intervention (the NEXit intervention) [11,17–19]. The trial was conducted simultaneously at all universities and colleges in Sweden (except one which participated in the pilot study). Students were emailed invitations over a 3-week period (October 23 to November 13, 2014). The trial was approved by the Regional Ethical Committee in Linköping, Sweden (Dnr 2014/217-31), and was prospectively registered (ISRCTN75766527).

A total of 1590 students were randomized (eligible due to being daily or weekly smokers), of which 827 were allocated to the intervention group and 763 to the control group (waiting list). Approximately 70% were female, and the majority were between 21 to 25 years old.

At 3-months after randomization, follow-up data was collected from 1502 students (94.5%, 1502/1590), loss to follow-up was due to participants not being contactable by phone. Follow-up rates did not differ significantly between groups and attrition analyses did not identify any systematic difference between those who did and did not complete follow-up.

The primary outcome measure in the NEXit trial was subjective reporting of prolonged abstinence, following the Russel standard definition [20], as not having smoked more than 5 cigarettes the past 8 weeks. The intervention was found to have a statistically significant effect on prolonged abstinence, with an OR of 2.05 (95% CI 1.57–2.67, P-value < .001). Prevalence of prolonged abstinence in the intervention group was 25.9% and in the control group 14.6%, giving an average treatment effect estimate of 11.3 percentage points absolute difference. For more information regarding the NEXit intervention and the RCT we refer the interested reader to published reports [11,17–19].

### Model

For this study on heterogenous treatment effects, we used the same logistic regression model used in the original group comparison analysis, with the same covariates and primary outcome. However, a Bayesian approach to coefficient inference was taken. This was done in order to: (1) calculate predictions using the joint posterior predictive distribution over coefficients, which may produce better predictions than approaches relying on point estimates [21]; (2) incorporate uncertainty regarding which coefficients to include in the model using shrinkage priors (see later description). Posterior distributions were calculated using Hamiltonian Monte Carlo (HMC) [22], a Markov chain Monte Carlo method particularly used in the probabilistic programming language STAN [23]. So called *horseshoe* priors were used for covariate coefficients, which favors shrinking coefficients towards zero, encoding an a-priori belief that single covariates do not dominate the odds of smoking cessation.

The full Bayesian model is presented in Eq 1, with priors as recommended by [24]. In the equation, the covariates are as follows: *G* = group allocation, *S* = sex, *Y* = the number of years smoked, *W* = the number of cigarettes smoked weekly, *D* = dependence according to Fagerström’s test for nicotine dependence, *U* = the amount of snuff used. Coefficients (except for the intercept) are given normal priors with variance defined by a global parameter *τ* and local parameters *λ*_1–6_. Half-Cauchy priors for *τ* and *λ*_1–6_ are set so that *τ* shrinks all coefficients towards zero and *λ*_1–6_ allow for individual coefficients to avoid this shrinkage. The scalar *τ*_0_ is defined by $\tau_{0}=\frac{p_{0}}{K-p_{0}}\times \frac{2}{\sqrt{N}}$, where *p*_0_ represents the number of covariates that a-priori are believed to be effectively non-zero, *K* the number of covariates, and *N* the number of records in the dataset. In our case, we replaced categorical variables (S, D and U in Eq 1) with dummy variables representing the levels of the original variables. This resulted in 11 columns in the dataset (ie. K = 11), and we decided to set *p*_0_ = 6, which equals the number of columns in our dataset divided by two (rounded up). The reasoning for this is that we do not believe that all covariates should be included in the model (or else we would not have included shrinkage priors), nor do we a-priori believe that none of the covariates should be included (or we would not have included them at all). Instead our best guess, prior to seeing any data, is to assume that half of them should remain. The inference is quite insensitive to the choice of *p*_0_, especially when the number of records available far outweighs the number of covariates, as it does in our case. A detailed discussion about this insensitivity can be found in [24].

We used precompiled STAN code supplied in the R package rstanarm. R version 3.5.3 and rstanarm 2.18.2 was used for all inference. HMC was used to collect 2000 samples from the joint posterior distribution for each iteration of the cross-validation procedure. The procedure outlined in this Methods section took approximately 20 minutes to run on a 2017 MacBook Pro.

$$\begin{array}{l} prolonged\;abstinence\;\sim \;Bernoulli\left(p\right)\\ \text{log}\left(\frac{p}{p-1}\right)=\beta_{0}+\beta_{1}G+\beta_{2}S+\beta_{3}Y+\beta_{4}W+\beta_{5}D+\beta_{6}U\\ \beta_{0}\;\sim \;t_{7}\left(0,\;2.5\right)\\ \beta_{1-6}|\tau ,\lambda_{1-6}\;\sim \;N\left(0,\;\tau \;\lambda_{1-6}\right)\\ \lambda_{1-6}\;\sim \;HalfC\left(0,1\right)\\ \tau \;\sim \;HalfC\left(0,\tau_{0}^{2}\right) \end{array}$$ (1)

Eq 1. Logistic regression model for smoking cessation using horseshoe priors.

### Predictive performance and cross-validation

In order to validly explore heterogeneous treatment effects, we have to make predictions that are accurate. If a model tells us that 15% of the individuals with access to a certain treatment will report prolonged smoking abstinence, then we also expect 15% of these individuals to actually have been abstinent, not 5% or 25%. We refer to a model’s capacity to produce accurate probabilities as the model’s predictive performance.

**Fig 1:**
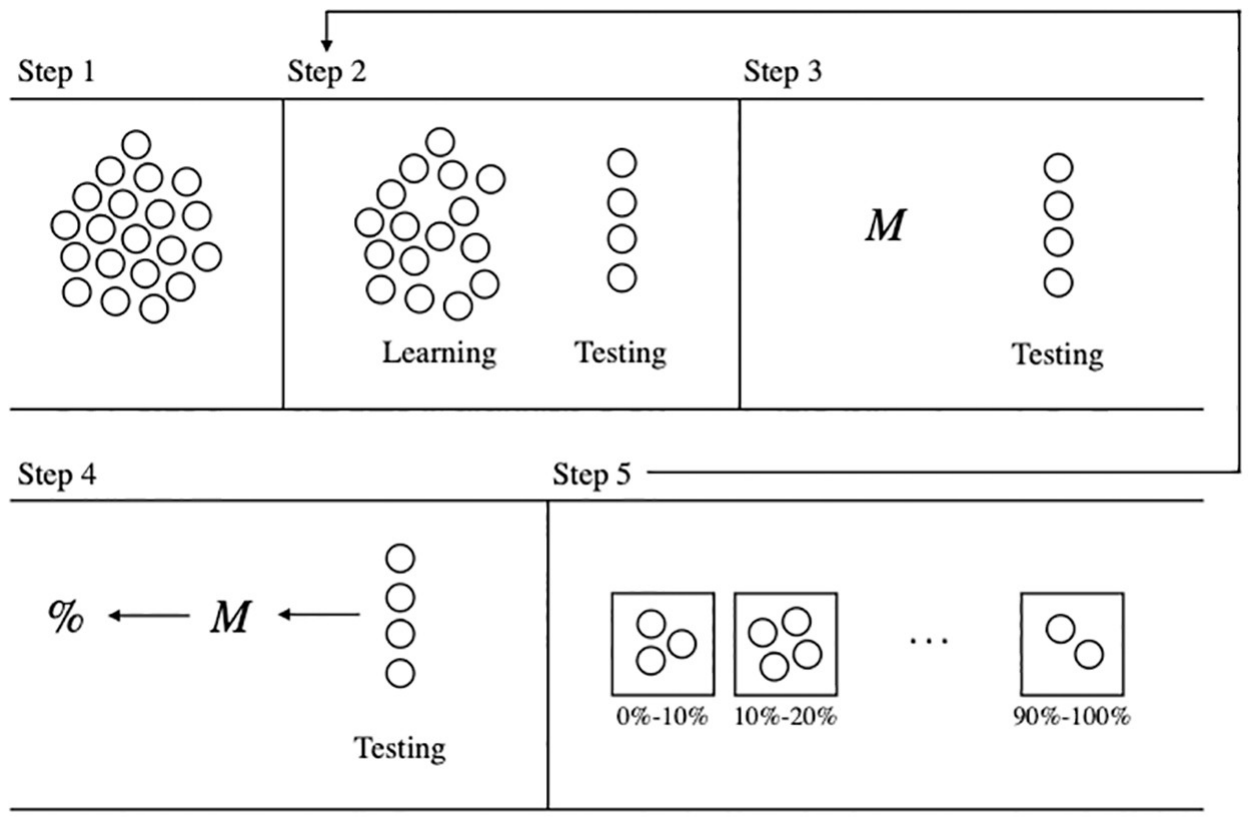
Cross-validation procedure to assess the performance of a prediction model.

A visualization of the procedure we use to assess predictive performance using a technique known as cross-validation is shown in Fig 1. Each circle in Step 1 represents a participant in the RCT. In Step 2, we randomly split the participants into two subsets, one which contains 90% of the participants, and one that contains the remaining 10%. We call these two subsets the *learning data* (the larger subset) and the *testing data* (the smaller subset).

Using the learning data, we run HMC to infer posterior distributions of the covariates in Eq 1, and thereby define a model *M* in Step 3. We do not involve the participants from the testing data when creating this model, but rather we treat them as if they were never part of the RCT at all. In Step 4, we input each participant withheld in the testing data into *M*, one at a time, and output a predicted probability that this individual will quit smoking. In Step 5, we place each participant from the testing data into a bin. The first bin is labelled 0%-10%, the next is labeled 10%-20%, and so on until we get the final bin labeled 90%-100%. We place each individual in the bin that corresponds to the prediction that we made for them, eg. if *M* predicted that an individual had a 15% probability of prolonged abstinence, then we place this individual in the bin labelled 10%-20%.

We then go back and repeat the procedure from Step 2 onwards in such a manner that each participant will be part of the testing data exactly once. This results in each participant being placed in one of the ten bins in Step 5.

Predictive performance of the model can be assessed by looking into the bins. The average prediction made within each bin should correspond to the actual ratio of occurrence of the outcome being predicted. For instance, assuming that we on average predicted a 15% probability of prolonged abstinence for the participants that we placed in the 10%-20% bin, then we expect 15% of these participants to actually have reported prolonged abstinence. We can make these calculations since we have follow-up data from the RCT, and a model’s predictive performance can be judged by how well the predictions are aligned with the empirical findings.

Note, when this procedure is operationalized it is common to use locally weighted scatterplot smoothing (lowess) in order to visually show a model’s predictive performance. Doing so allows us to examine the model’s performance across a range of predicted values rather than within a set of predefined bins [25,26]. The visualization using bins is however still useful to understand the concept behind what we refer to as predictive performance, and one may think about using lowess as using an infinite number of bins.

### Individual treatment effects

Estimates of individual level treatment effects will be calculated during the cross-validation procedure described earlier. In Step 4 (see Fig 1), we predict the probability of prolonged abstinence (Russel standard [20], no more than 5 cigarettes the past week) for each individual given both the intervention and control setting. By subtracting these two probabilities from each other we get the individual level effect of the intervention in terms of absolute difference in percentage points. For instance, if an individual is predicted to have a 15% probability of prolonged abstinence if they are given access to the intervention, and a 5% without access, then the probability of prolonged abstinence increases with 10 percentage points if the individual has access to the intervention. In this context, access to the intervention is defined as being offered the intervention, as this reflects the intervention group in the NEXit trial [11].

## Results

### Predictive performance

**Fig 2:**
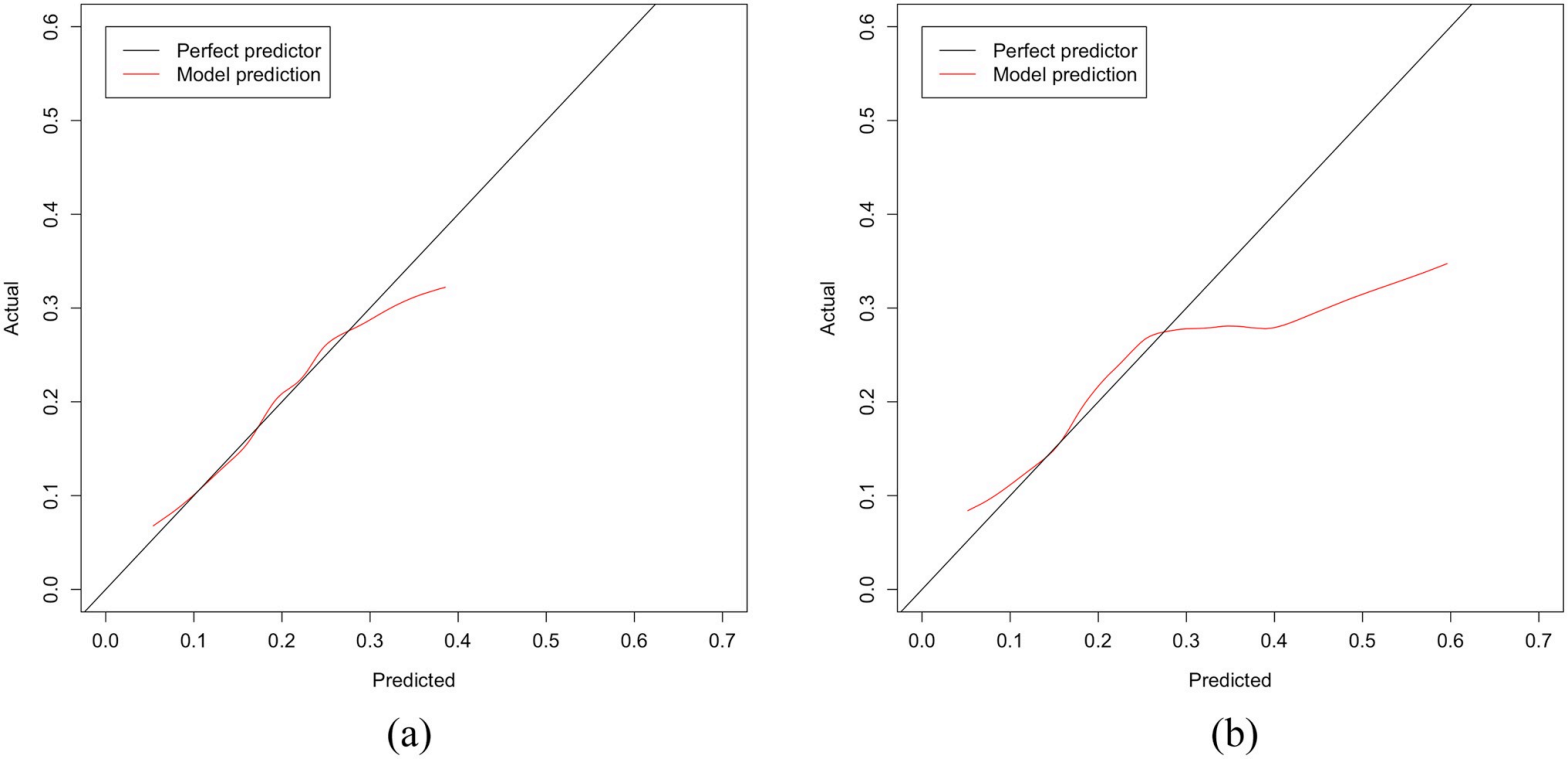
The red line will overlap the black line when predictions are accurate. (a) Predictive performance of model in Eq 1 on the withheld testing data during cross-validation using Bayesian inference. (b) Predictive performance of the same model using maximum likelihood estimates.

Fig 2(a) illustrates the predictive performance of the model in Eq 1 on the withheld testing data during cross-validation. The black line represents a perfect model which predicts probabilities that are accurate to what has been observed empirically, the closer we get to this line the better. The red line represents the model learnt using the procedure outlined in Fig 1. The line is drawn using lowess, and as described earlier, this can be understood as using an infinite number of bins rather than choosing a fixed number. The model’s performance is close to the black line; thus, it tells us that when the model predicts that an individual has a certain probability of prolonged abstinence, then this is also the probability we should expect to see empirically.

For reference, we ran the procedure in Fig 1 using the model used in the original publication [11], ie. the same model as in Eq 1 but using maximum likelihood estimates rather than Bayesian inference. Fig 2(b) visualizes the predictive performance of this model. It is clear that this model produces erroneous predictions, as the red line indicates that optimistic predictions are being made, for instance some cases are predicted to have a 60% probability of prolonged abstinence despite this not being observed empirically. This should serve as a reminder that while it is common to report maximum likelihood estimates of coefficients when contrasting groups in trials, it is not necessarily the case that these models have good predictive performance.

### Conditional average treatment effects

**Fig 3:**
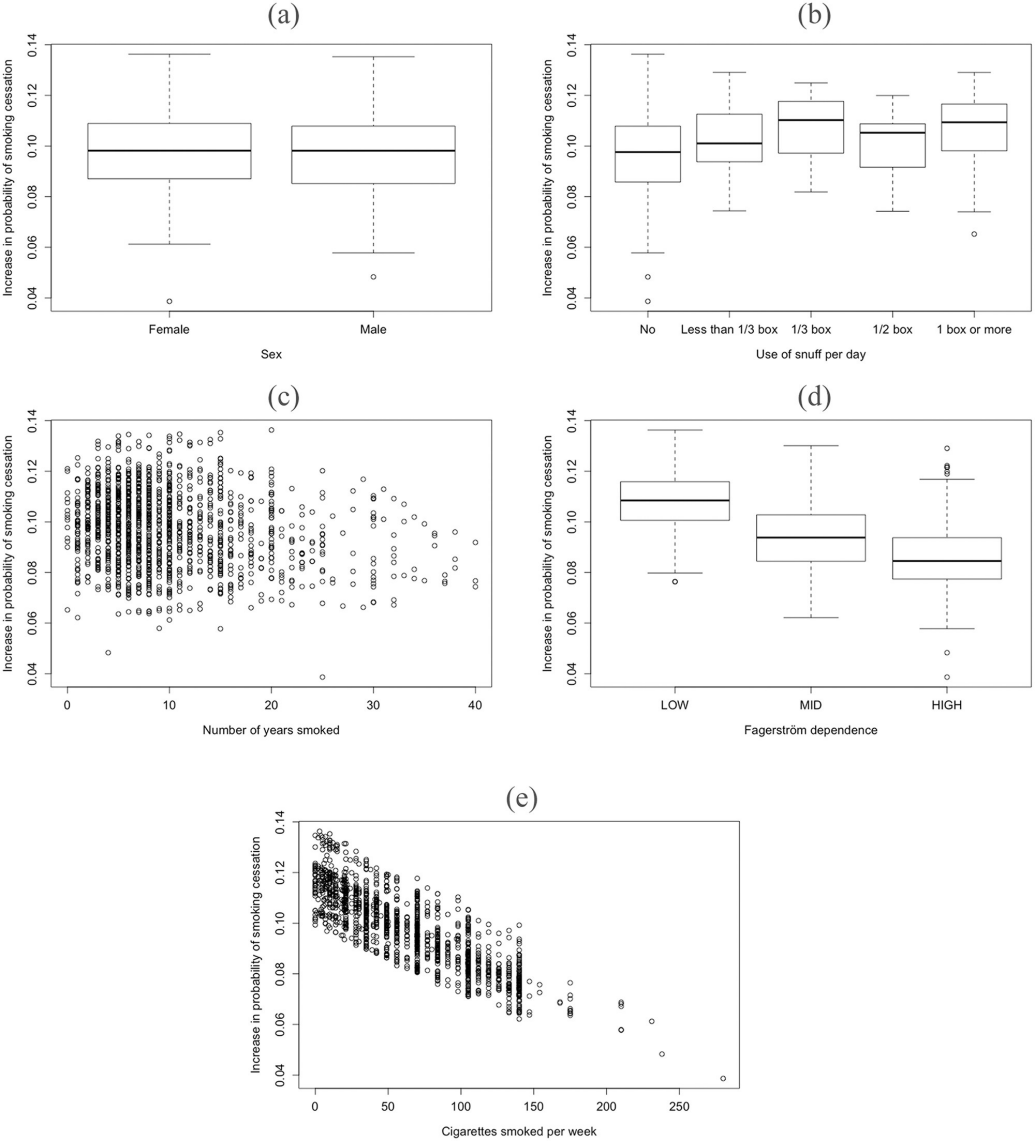
Heterogeneous treatment effects of the NEXit smoking cessation intervention. (a-c) No heterogeneity is apparent with respect to sex, the use of snuff, nor the number of years smoked. (d-e) The NEXit intervention is less effective among those with stronger nicotine dependence and those who smoke more per week.

In Fig 3(a)–3(e) we have plotted the absolute difference in predicted prolonged abstinence (no more than 5 cigarettes the past 8 weeks) against each of the covariates used in the model in Eq 1. In Fig 3(a) we can see that the absolute difference in predicted prolonged abstinence among female students are similar to those among male students, on average 10 percentage point absolute difference between being offered the intervention and not offered the intervention. Thus, there does not seem to be any heterogeneity in treatment effects with respect to sex. Similarly, there does not seem to be much heterogeneity with respect to the use of snuff (Fig 3(b)) or the number of years smoked (Fig 3(c)).

Heterogeneity does however seem to exist when taking into consideration students’ nicotine dependence (Fagerström’s test for nicotine dependence), as can be seen in Fig 3(d). The absolute difference in predicted probability of prolonged abstinence is on average approximately 11 percentage points for those with low dependence, but closer to 9 percentage points for those with high dependence. The ranges are quite wide for these estimates, yet there does seem to exist a trend of reduced effect of the intervention as the severity of dependence increases. Put differently, the model predicts that the NEXit intervention is more effective among university students with low dependence to nicotine than among those with high dependence. This should however not be interpreted as a causal connection, thus we do not suggest that changing the nicotine dependence for a specific student will change the effectiveness of the intervention for this individual. Instead, the findings are observational in nature: among those with higher nicotine dependence the NEXit intervention was predicted to be less effective. A similar narrative can be given when looking at the number of cigarettes smoked per week (Fig 3(e)). As the number of cigarettes smoked increases, the absolute difference of predicted prolonged abstinence between intervention and control decreases, suggesting that the intervention is less effective among students who smoke more.

## Discussion

When studying heterogenous treatment effects of the NEXit intervention, we found that the intervention’s effect on university students was reduced among those with stronger nicotine dependence and among those who smoked more cigarettes per week. This should not be interpreted as a causal connection between nicotine dependence and the effectiveness of the NEXit intervention, but rather an associational one which we may be able to exploit when deciding which intervention to allocate to which subgroup in a population.

The findings from this study questions the purpose of future developments of digital smoking cessation aids. As prevalence rates of smoking drops in a population, it may be the case that among those who still smoke a great majority have a stronger dependence to nicotine. If this is the case, then the results enclosed suggests that text messaging interventions may not be the right tool for further reduction of prevalence rates of smoking. However, this is only a hypothetical scenario which needs to be supported by future research and data aggregation.

The NEXit intervention was more supportive of smoking cessation among students who had not yet developed a strong nicotine dependence (defined by Fagerström’s test for nicotine dependence). Thus, it may be an ideal tool to make available to university students to help them quit before they develop a stronger dependence. As data in Sweden suggest that smoking is still prevalent among young individuals, a digital intervention which is easily scaled to a national level could protect from increasing prevalence of smoking in the future. However, any decision of full-scale dissemination should be based on careful consideration of other factors, including other types of interventions, costs, time and resources.

There is a need to conduct heterogeneous treatment effect analyses of other types of smoking cessation interventions in order to get a better picture of which interventions are effective for which individuals. For instance, it was found that a web-based smoking cessation program as a supplement to nicotine patch therapy was effective in supporting smoking cessation after 12 weeks (OR = 1.33 of 10-week abstinence; 95% CI = 1.13–1.57; P-value < .001) [27], however, so was a digital intervention (using email, web-pages, interactive voice response, and text messaging) without the use of nicotine replacement after 12 weeks (OR = 2.93 of 7-day abstinence, 95% CI = 1.67–5.14, P < .001) [27]. Results from both trials may be masking important heterogenous treatment effects, which could be compared to those enclosed, in order to better understand which individuals benefit the most (and least) from different smoking cessation interventions.

### Limitations

The results of the analyses included herein should be understood under several conditions. The population under consideration are university students, thus our findings may not be generalizable to other subpopulations or the general population as a whole. Also, readers should recall that in all RCTs we are formally testing the randomization component rather than one treatment against another, as individuals may decide to adhere and engage with an intervention at their own discretion. Thus, while being randomized may sometimes be a good proxy to treatment, the estimates in this analysis should be strictly understood as comparing access to the intervention rather than use of it.

### Conclusions

Using methods common in the machine learning field, we were able to get reliable estimates of heterogeneous treatment effects of the NEXit intervention. The findings suggest that subpopulations among university students were predicted to react differently to the NEXit intervention, and this was not evident from the original analyses when the intervention and control groups were compared directly. Thus, the heterogenous treatment effects were masked in the original analysis [11].

This study is the first of its kind with respect to text messaging interventions for smoking cessation among university students. We recommend other researchers to pursue similar studies in order to strengthen the evidence and corroborate results. It should be noted that in trials where no average treatment effect was found, the exploration of heterogenous treatment effects may reveal if there are subgroups for which the effect is being masked by the overall treatment effect. We also need a better understanding of heterogeneous treatment effects of other available interventions, eg. face-to-face and group counselling, in order to decide which individuals should be targeted by future intervention developments.
